# Transcriptomic Profiling of Adipose Tissues in Sujiang Pigs Reveals Candidate Genes Associated with Tissue-Specific Fat Deposition

**DOI:** 10.3390/life16061024

**Published:** 2026-06-18

**Authors:** Huizhen Gao, Shubin Zhu, Ligang Ni, Feixiang Cao, Pan Xu

**Affiliations:** 1 School of Modern Animal Husbandry, Jiangsu Agri-Animal Husbandry Vocational College, Taizhou 225300, China; 2 School of Artificial Intelligence, Jiangsu Agri-Animal Husbandry Vocational College, Taizhou 225300, China

**Keywords:** Sujiang pig, subcutaneous and visceral adipose tissue, RNA-Seq, lipid metabolism, inflammation

## Abstract

In addition to its role in energy storage, adipose tissue contributes substantially to energy metabolism, endocrine regulation, and inflammatory processes. Sujiang pigs, a hybrid breed approved by the National Livestock and Poultry Genetic Resources Committee of China as a new national breed in 2013, possess a genetic predisposition for substantial fat deposition, making them an ideal model for investigating the mechanisms underlying adipose tissue accumulation. In this study, back fat (BF; subcutaneous adipose tissue), greater omentum (GOM; visceral adipose tissue), and mesenteric adipose tissue (MAD; visceral adipose tissue) were collected from three 6-month-old male Sujiang pigs for RNA-seq analysis. Comparative analyses identified 3005 differentially expressed genes (DEGs) between BF and GOM, 975 DEGs between BF and MAD, and 892 DEGs between GOM and MAD. To validate the reliability of the sequencing data, five DEGs were randomly selected for RT-qPCR verification. The DEGs were further subjected to Gene Ontology (GO) and Kyoto Encyclopedia of Genes and Genomes (KEGG) enrichment analyses. By integrating protein–protein interaction (PPI) networks with bioinformatics analyses, we identified candidate genes potentially associated with lipid metabolism (e.g., WNT9A, WNT5A, and PDGFRA) and inflammatory responses in adipose tissue (e.g., CSF1R, C1QB, and CD4). These findings indicate potential molecular differences between porcine visceral and subcutaneous adipose tissues and may serve as a reference for further studies on the molecular regulation of adipose tissue metabolism.

## 1. Introduction

The growth and metabolism of adipose tissue are critical factors influencing both the growth rate and meat quality of pigs [1,2]. Adipose tissue comprises adipocytes, non-adipocyte cells, connective tissue, blood vessels, and nerve tissue, and participates in essential biological processes such as energy metabolism, neuroendocrine regulation, and immune function [3]. Based on anatomical distribution, adipose tissue is classified into subcutaneous adipose tissue (SAT) and visceral adipose tissue (VAT); VAT is located in the mesentery, omentum, and surrounding internal organs, whereas SAT is predominantly found beneath the skin [4,5]. VAT and SAT differ not only in structural characteristics but also in metabolic profiles and associated physiological functions, including the uptake of free fatty acids and triglycerides, lipid metabolic activity, glucose uptake capacity, and insulin sensitivity [6].

To date, research on the physiological functions of adipose tissue has largely relied on rodent models. In contrast, few studies have directly compared VAT and SAT under normal physiological conditions [7]. Currently, mechanistic studies on differential fat deposition in pigs primarily emphasize subcutaneous and intramuscular fat [8,9,10,11]. In contrast, research comparing VAT and SAT in pigs remains limited. The Sujiang pig is an important cultivated breed in China, developed through artificial selection from Jiangquhai, Fengjing, and Duroc pigs. It is characterized by rich flavor compounds, excellent meat quality, and strong fat deposition ability. However, no reports have identified regulatory genes associated with differences in visceral and subcutaneous fat deposition in Sujiang pigs.

Herein, we collected VAT and SAT from Sujiang pigs under normal conditions for comparative analysis. Using RNA-seq technology, we performed transcriptome sequencing to identify differentially expressed genes between VAT and SAT, from which key regulatory genes were screened to elucidate the molecular mechanisms underlying site-specific fat deposition in Sujiang pigs. This work provides a theoretical foundation for further exploration of fat deposition and metabolism.

## 2. Materials and Methods

### 2.1. Animals and Sample Collection

Sujiang pigs were obtained from Jiangsu Sujiang Breeding Pig Co., Ltd., Taizhou, China. The animals were housed in indoor pens with an average space allowance of 2–3 m^2^ per pig. Environmental conditions were monitored continuously to ensure consistency throughout the feeding period, and the pigs had *ad libitum* access to food and water. The experimental diet, primarily composed of corn and soybean meal, was formulated to meet the crude protein, trace mineral, and vitamin requirements specified by the National Research Council (NRC 1998) for different growth stages. Three healthy, 6-month-old male Sujiang pigs with similar body weights were randomly selected from the same feeding group to serve as independent biological replicates. Prior to slaughter, the selected pigs were fasted for 12 h. Slaughtering was performed at a facility separate from the farm, using electric stunning to minimize pain and stress. Within 30 min of exsanguination, samples of back subcutaneous fat (BF), greater omentum (GOM), and mesenteric adipose tissue (MAD) were collected, immediately snap-frozen in liquid nitrogen, and stored at −80 °C for subsequent analysis. This study strictly adhered to the Regulations on the Administration of Laboratory Animals. The experimental protocol was approved by the Ethics Committee of Jiangsu Vocational College of Agriculture and Animal Husbandry Science on 12 January 2024 (approval number: SYXK(Su)IACUC2024-JB02).

### 2.2. Total RNA Extraction, Library Construction, and Sequencing

Total RNA was extracted from tissue samples using the Total RNA Extractor kit (Sangon Biotech, Shanghai, China; catalog no. B511311) according to the manufacturer’s instructions. RNA purity was assessed using an SMA4000 micro-spectrophotometer (Merinton), and concentration was determined with the Qubit 2.0 RNA Assay Kit (Invitrogen; catalog no. Q32866). RNA integrity and potential contamination were evaluated by 1.0% agarose gel electrophoresis. Only total RNA samples meeting the quality control criteria were used for subsequent library construction and transcriptome sequencing.

mRNA was enriched using Oligo(dT) magnetic beads, fragmented, and reverse-transcribed into cDNA using random primers. Following purification, the fragments underwent end repair, poly(A) tail addition, and ligation of sequencing adapters. Size-selected fragments were then PCR-amplified to generate the final sequencing libraries. Sequencing was performed on an Illumina NovaSeq 6000 platform by Sangon Biotech (Shanghai) Co., Ltd.

### 2.3. Sequencing Data Processing and Identification of Differentially Expressed Genes

Paired-end 150 bp (PE150) sequencing was performed on the Illumina NovaSeq 6000 platform. Raw sequencing reads were assessed for quality using FastQC (version 0.11.2). Trimmomatic (version 0.36) was employed to remove low-quality bases and adapter sequences to obtain high-quality clean reads. These clean reads were aligned to the pig reference genome (Sscrofa11.1) using HISAT2 (version 2.0). Read alignment statistics were generated using RSeQC (version 2.6.1). Qualimap (version 2.2.1) was used to evaluate read distribution uniformity and genomic structural features, while BEDTools (version 2.26.0) was used for gene coverage statistical analysis. Gene read counts were obtained using the featureCounts function from the Subread package. Differential expression analysis was performed using DESeq2 with the design = ~condition (adipose tissue). Additionally, StringTie (version 1.3.3b) was used to quantify gene expression levels in transcripts per million (TPM) for visualization purposes. Differentially expressed genes (DEGs) were identified using the criteria of *q*-value ≤ 0.05 and |log_2_FoldChange| ≥ 1.

### 2.4. Real-Time Quantitative PCR Validation

To validate the RNA-seq results, five DEGs—C1QB, CD163, C1QC, TIMP1, and TFPI2—were randomly selected for quantitative real-time PCR (RT-qPCR) validation. GAPDH served as the internal reference gene (primer sequences are provided in Table 1). The 20 µL qRT-PCR reaction mixture contained 2 µL cDNA template, 0.4 µL each of forward and reverse primers, 10 µL SYBR Green qPCR Master Mix, and 7.2 µL nuclease-free water. Amplification was performed under the following conditions: initial denaturation at 95 °C for 3 min, followed by 45 cycles of 95 °C for 15 s and 60 °C for 30 s. Relative gene expression levels were calculated using the 2^−ΔΔCt^ method.

### 2.5. Differentially Expressed Gene GO and KEGG Pathway Enrichment Analysis

GO functional annotation and Kyoto Encyclopedia of Genes and Genomes (KEGG) pathway enrichment analyses of the DEGs were performed using a significance threshold of *p* < 0.05.

### 2.6. Candidate Gene Prediction

Protein–protein interactions (PPIs) of the selected genes were analyzed using the STRING database (https://cn.string-db.org/, accessed on 10 May 2025) to construct a protein interaction network. Subsequently, candidate genes were identified using Cytoscape software (version 3.10.1).

## 3. Results

### 3.1. Sequencing Data Quality Control

In this study, nine cDNA libraries were constructed from subcutaneous BF, GOM, and MAD tissues of Sujiang pigs using the Illumina sequencing platform. These libraries were designated as 1-SJ-BF, 2-SJ-BF, 3-SJ-BF, 1-SJ-GOM, 2-SJ-GOM, 3-SJ-GOM, 1-SJ-MAD, 2-SJ-MAD, and 3-SJ-MAD. Each library yielded >6 Gb of clean data. Alignment to the pig reference genome was performed using HISAT2, achieving an average mapping rate of 97% and a unique mapping rate of >94%. Sequencing quality statistics are presented in Table 2. The raw reads for all samples exceeded 45,328,520, and after quality control, the number of valid sequences exceeded 43,207,246 for each sample. Across the nine sequencing libraries, GC content ranged from 49.94% to 53.35%, and Q20 and Q30 values were consistently above 98.23% and 93.91%, respectively. These metrics indicate that the sequencing data are of high quality and suitable for downstream analyses (Table 2).

### 3.2. Sequence Alignment Analysis

The quality-controlled reads were aligned to the pig reference genome, and the alignment statistics are presented in Table 3. The overall alignment rate across all samples exceeded 97.35%, and the proportion of reads uniquely mapped to the gene set was greater than 94.72%. These high alignment rates, coupled with consistent mapping performance among samples, indicate that the sequencing data are comparable across the experimental groups (Table 3).

### 3.3. Screening and Analysis of Differentially Expressed Genes

**Figure 1:**
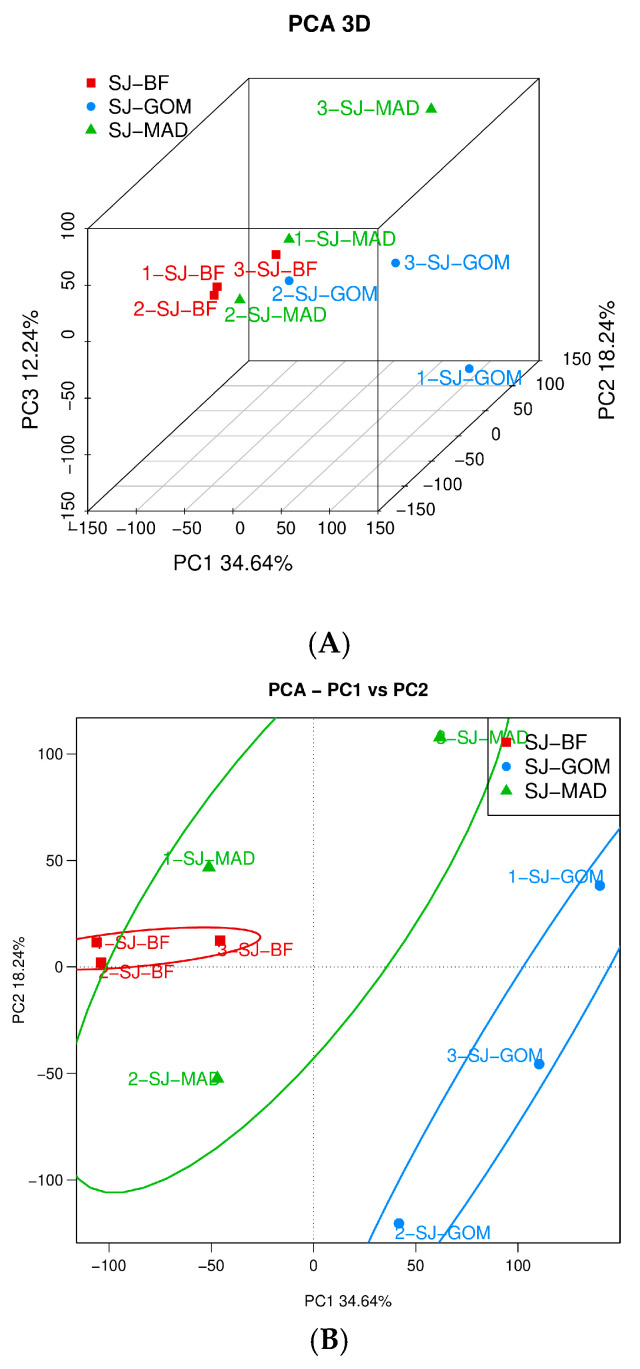
PCA results for BF, GOM, and MAD. Note: (**A**) Three-dimensional PCA scatter plot showing the spatial distribution of all samples along PC1, PC2, and PC3, which account for 34.64%, 18.24%, and 12.24% of the total variance, respectively. BF (red squares), MAD (green triangles), and GOM (blue circles) are distinguished by both color and shape; (**B**) Two-dimensional PCA scatter plot, representing the projection (top view) of the 3D plot in (**A**) onto the PC1-PC2 plane, to better visualize the group separation along the first two principal components. The color, shape, and grouping of samples are identical to those in (**A**).The scales on all axes represent relative distances among samples and do not have absolute meaning. Each point corresponds to one sample, and samples within the same group are shown in the same color.

DEGs were identified using the criteria of *q* Value ≤ 0.05 and |log_2_FoldChange| ≥ 1. Using the vegan package (version 2.0-10) in R, a three-dimensional principal component analysis (PCA) was performed on BF, GOM, and MAD samples. The results showed that PC1, PC2, and PC3 explained 34.64%, 18.24%, and 12.24% of the total variance, respectively (Figure 1A). The PCA plot of PC1 versus PC2 (Figure 1B) showed that the three replicates of the same tissue clustered together, indicating good intra-group reproducibility. The BF group was located on the left, the MAD group in the center-left upper area, and the GOM group on the right. The three groups were completely separated without overlap, suggesting that the differences in gene expression between tissues were substantially greater than the variations caused by biological replicates within groups, thereby confirming the reliability of the experimental grouping.

**Figure 2:**
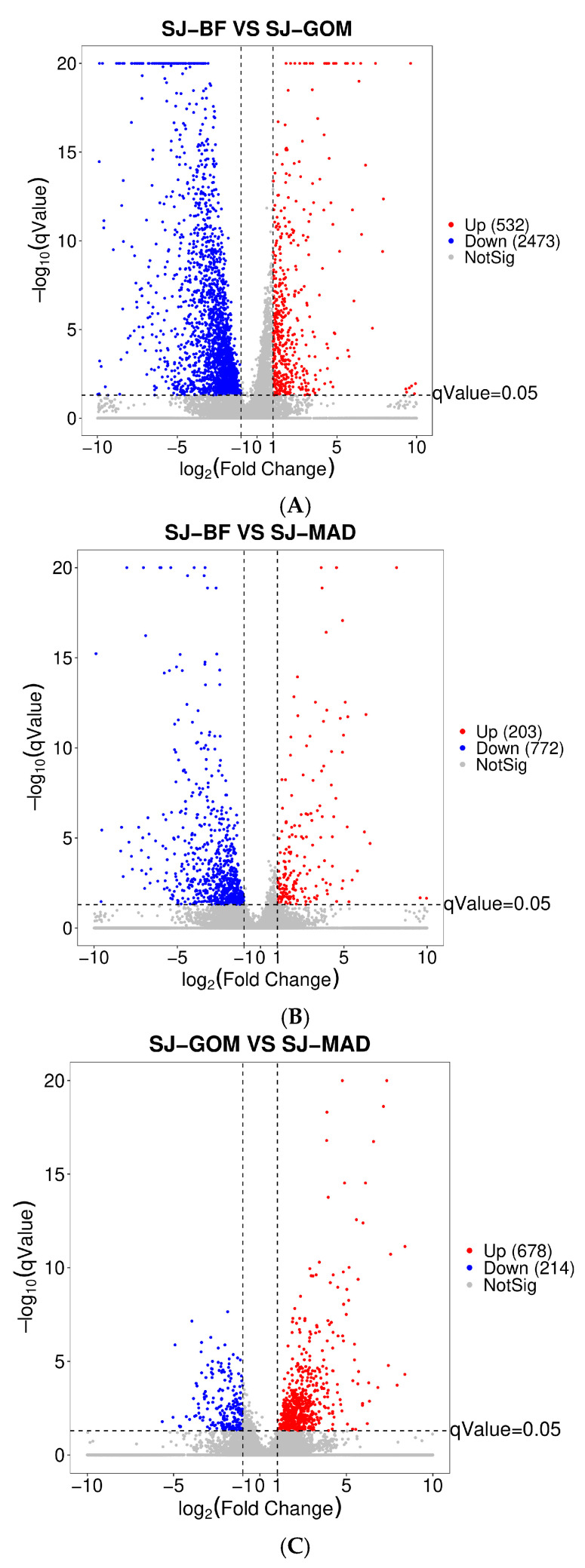
Volcano plot of differentially expressed genes between groups. Note: (**A**) Volcano plot showing differentially expressed genes in the SJ-BF vs. SJ-GOM comparison. The x-axis indicates log_2_(Fold Change), and the y-axis represents −log_10_(qValue). Red, blue, and gray dots correspond to significantly upregulated, significantly downregulated, and non-significantly expressed genes, respectively, with *q* Value = 0.05 as the significance threshold; (**B**) Volcano plot of differentially expressed genes from the SJ-BF vs. SJ-MAD pairwise comparison, with axes, gene color coding and significance threshold identical to panel (**A**); (**C**) Volcano plot displaying differentially expressed genes identified in the SJ-GOM vs. SJ-MAD comparison, with axes, gene color coding and significance threshold identical to panel (**A**).

**Figure 3:**
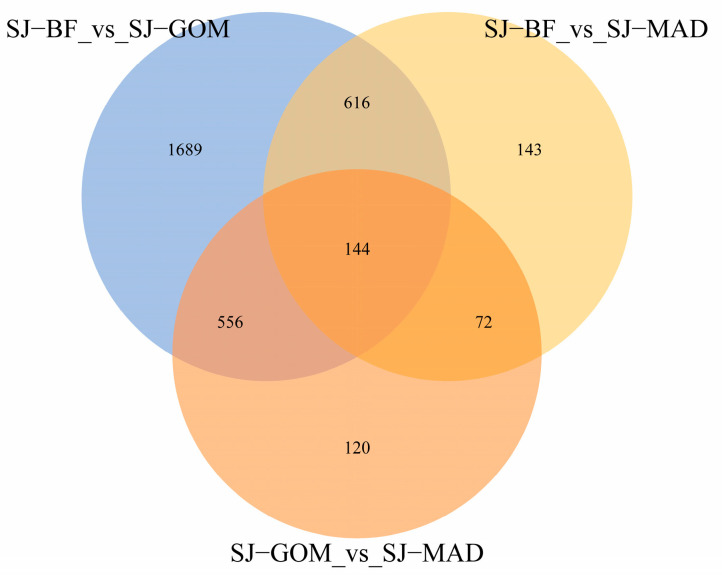
Differential gene Venn diagram. Note: Different comparison groups are represented by different colors, and the numbers in the figure represent the number of differentially expressed genes that are unique or shared. The overlapping areas represent the number of differentially expressed genes shared among different comparison groups, while the non-overlapping areas represent the number of differentially expressed genes unique to each comparison group.

The analysis identified a total of 3005 DEGs between the BF and GOM groups, comprising 532 upregulated and 2473 downregulated genes (Figure 2A); 975 DEGs between the BF and MAD groups, including 203 upregulated and 772 downregulated genes (Figure 2B); and 892 DEGs between the GOM and MAD groups, with 678 upregulated and 214 downregulated genes (Figure 2C). The top 10 differentially expressed genes are listed in Supplementary Tables S1–S3. The R VennDiagram package was employed to analyze overlapping DEGs across the three pairwise comparisons. As shown in the Venn diagram (Figure 3), the comparisons SJ-BF vs. SJ-GOM and SJ-BF vs. SJ-MAD shared 760 co-expressed genes; SJ-BF vs. SJ-GOM and SJ-GOM vs. SJ-MAD shared 700 co-expressed genes; SJ-GOM vs. SJ-MAD and SJ-BF vs. SJ-MAD shared 216 co-expressed genes; and all three comparisons shared 144 co-expressed genes. The SJ-BF vs. SJ-GOM, SJ-GOM vs. SJ-MAD, and SJ-BF vs. SJ-MAD comparisons contained 1689, 120, and 143 unique DEGs, respectively.

### 3.4. qRT-PCR Validation

**Figure 4:**
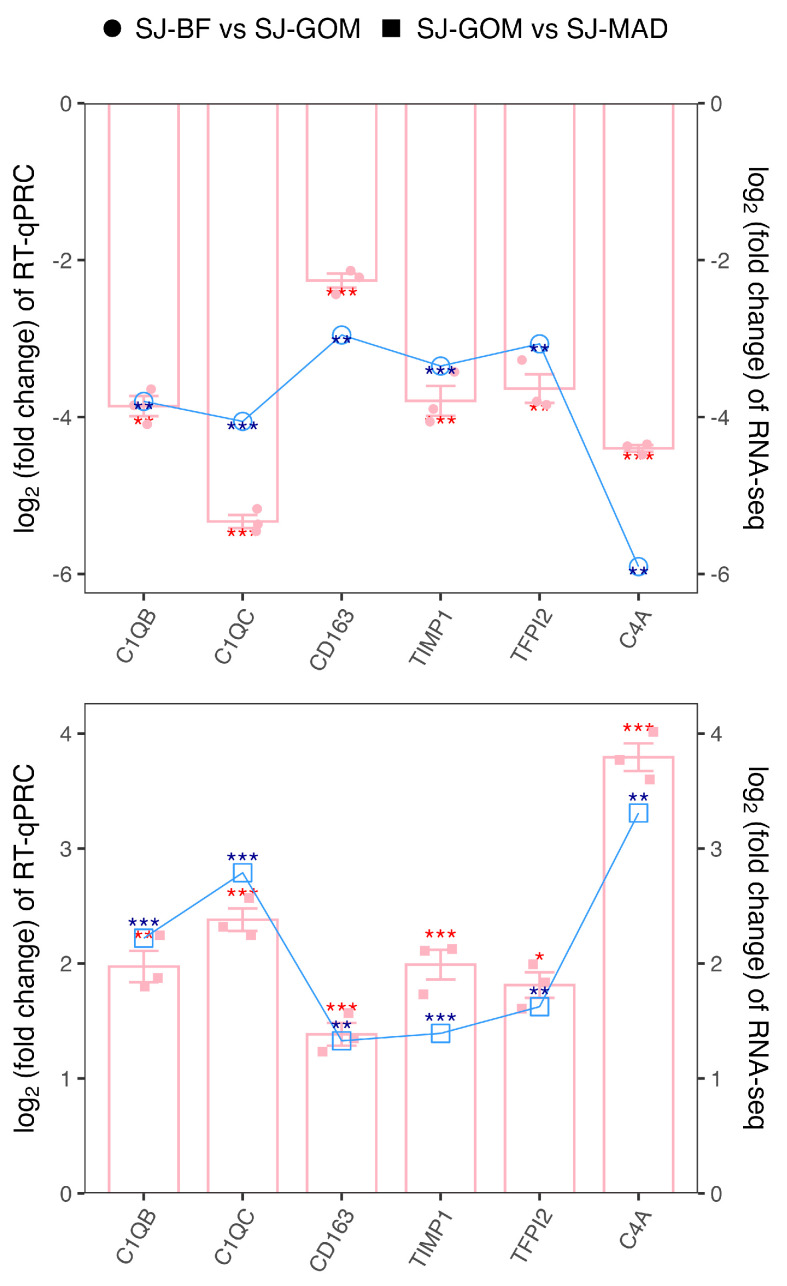
RT-qPCR verification results of DEGs. Note: The images show the comparison results of the BF-GOM and GOM-MAD groups, respectively. The left axis shows the log_2_ (fold change) of the RT-qPCR results, and the right axis shows the log_2_ (fold change) of the RNA-seq data. The pink bars represent the RT-qPCR data, the blue line with circles represents the RNA-seq results of BF-GOM, and the blue line with squares represents the RNA-seq results of GOM–MAD. Red asterisks indicate statistical significance of gene expression differences in RT-qPCR, and blue asterisks indicate statistical significance of gene expression differences in RNA-seq. * *p* < 0.05 ** *p* < 0.01 *** *p* < 0.001.

Six DEGs were randomly selected to validate the RNA-seq results using RT-qPCR. The results showed that the fold changes of the six genes in the RT-qPCR and RNA-seq datasets exhibited consistent trends (Figure 4), indicating the reliability of the RNA-seq results.

### 3.5. GO and KEGG Pathway Enrichment Analysis of Differentially Expressed Genes

#### 3.5.1. GO Enrichment Analysis

**Figure 5:**
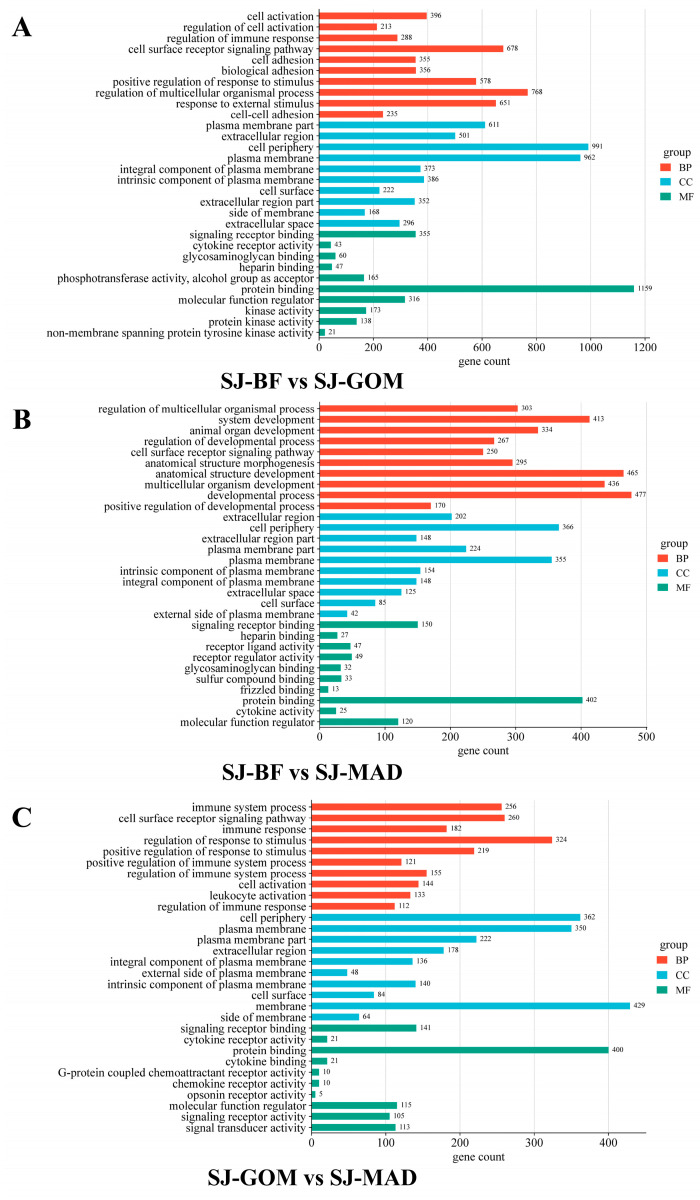
GO enrichment analysis results. Note: (**A**) GO enrichment analysis of DEGs from the SJ-BF vs. SJ-GOM comparison. The vertical axis displays enriched GO term names, and the horizontal axis shows the count of enriched genes. Red, blue and green bars represent biological process (BP), cellular component (CC), and molecular function (MF) categories, respectively; (**B**) GO enrichment analysis of DEGs from the SJ-BF vs. SJ-MAD pairwise comparison, with axis settings, color coding for GO categories and plotting criteria identical to panel (**A**); (**C**) GO enrichment analysis of DEGs identified in the SJ-GOM vs. SJ-MAD comparison, with axis settings, color coding for GO categories and plotting criteria identical to panel (**A**).

GO enrichment analysis was used to annotate the differentially expressed genes. The top 10 GO terms for BP, CC, and MF in each comparison group are shown (Figure 5A–C). The results of the GO enrichment analysis are as follows:

Among the DEGs identified between BF and GOM, 1688 GO terms reached statistical significance (*p* < 0.05). The majority (92.4%) were classified as biological processes, whereas molecular functions and cellular components accounted for 3.7% and 3.9%, respectively. These DEGs were mainly involved in regulatory biological processes, including cell activation, cell surface receptor signaling, regulation of multicellular organismal processes, cytokine production, and broader biological regulation.

In the BF versus MAD comparison, 1059 GO terms were significantly enriched (*p* < 0.05). These were predominantly associated with the regulation of developmental processes, cell surface receptor signaling, and the regulation of multicellular organismal processes.

Between GOM and MAD, 897 GO terms reached enrichment significance (*p* < 0.01). Of these, 805 were biological processes (92.4%), 52 were molecular functions (4.9%), and 40 were cellular components (3.7%). The enriched terms were primarily immune-related, covering immune system processes, cell surface receptor signaling, immune response, inflammatory response, and the regulation of multicellular organismal processes.

#### 3.5.2. KEGG Enrichment Analysis

Differentially expressed genes in adipose tissue were analyzed for pathway enrichment using the KEGG database.

**Figure 6:**
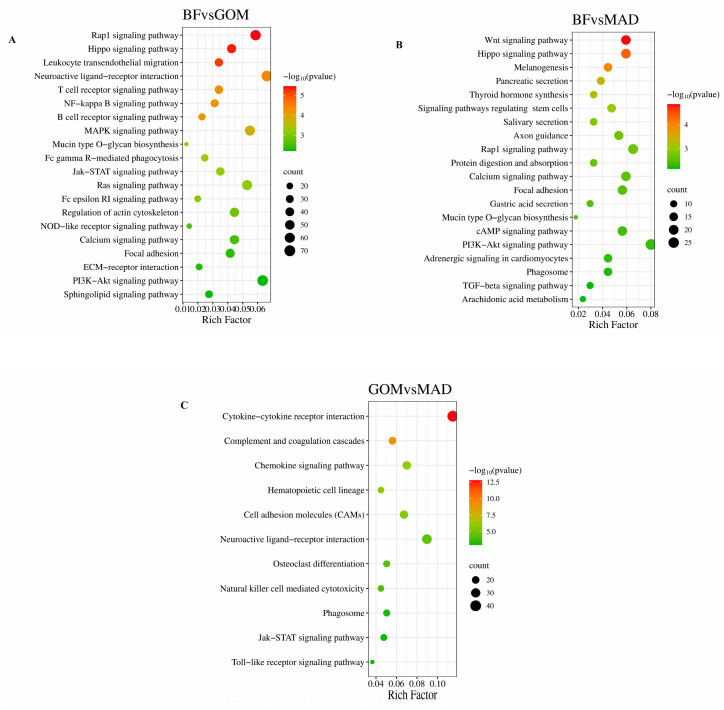
KEGG pathway analysis. Note: (**A**) KEGG enrichment bubble plot of DEGs from the SJ-BF vs. SJ-GOM comparison. The horizontal axis shows Rich factor, the vertical axis displays pathway names. Dot size corresponds to the number of enriched differential genes, and dot color represents −log_10_(*p* value) to reflect enrichment significance; (**B**) KEGG enrichment bubble plot of DEGs from the SJ-BF vs. SJ-MAD pairwise comparison, with axis definitions, dot size and color coding consistent with panel (**A**); (**C**) KEGG enrichment bubble plot of DEGs identified in the SJ-GOM vs. SJ-MAD comparison, with axis definitions, dot size and color coding consistent with panel (**A**).

KEGG enrichment analysis of differentially expressed genes between BF and GOM mapped to 242 pathways, with 32 pathways significantly enriched (*p* < 0.05). These genes were significantly enriched in pathways associated with lipid metabolism, including the Rap1 signaling pathway, Hippo signaling pathway, mucin-type O-glycan biosynthesis, regulation of the actin cytoskeleton, calcium signaling pathway, and sphingolipid signaling pathway, among others (Figure 6A).

KEGG enrichment analysis of differentially expressed genes between BF and MAD identified 182 signaling pathways, of which 27 were significantly enriched (*p* < 0.05). These primarily included the Wnt signaling pathway, Hippo signaling pathway, pancreatic secretion, thyroid hormone synthesis, signaling pathways regulating pluripotency of stem cells, Rap1 signaling pathway, calcium signaling pathway, cAMP signaling pathway, and other lipid metabolism-related pathways (Figure 6B).

By intersecting the significantly enriched pathways from the BF vs. GOM and BF vs. MAD comparisons, we identified shared pathways representing differentially expressed genes between VAT and SAT. These pathways were predominantly related to immunity and lipid metabolism, including the Rap1 signaling pathway, Hippo signaling pathway, calcium signaling pathway, cAMP signaling pathway, and Wnt signaling pathway. A total of 54 differentially expressed genes were annotated within these pathways (Table 4).

Differentially expressed genes between GOM and MAD were enriched in 187 KEGG signaling pathways, with 11 significantly enriched (*p* < 0.05). These were primarily associated with immune regulation, including cytokine-cytokine receptor interaction, complement and coagulation cascades, chemokine signaling pathway, hematopoietic cell lineage, cell adhesion molecules (CAMs), natural killer cell-mediated cytotoxicity, and phagosome (Figure 6C). This suggests that differentially expressed genes distinguishing VAT depots are mainly involved in immune and inflammatory responses, encompassing a total of 22 genes (Table 5).

### 3.6. Differentially Expressed Gene Protein–Protein Interactions (PPI) Analysis and Candidate Gene Identification

**Figure 7:**
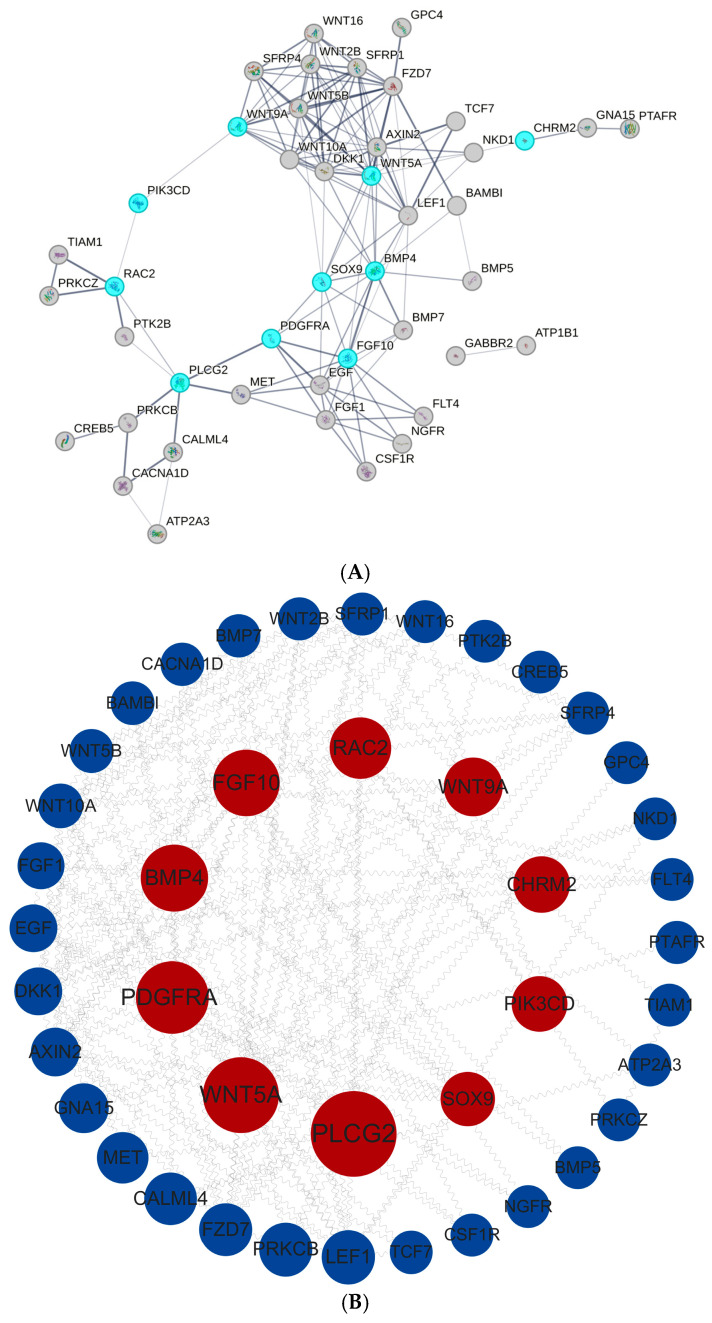

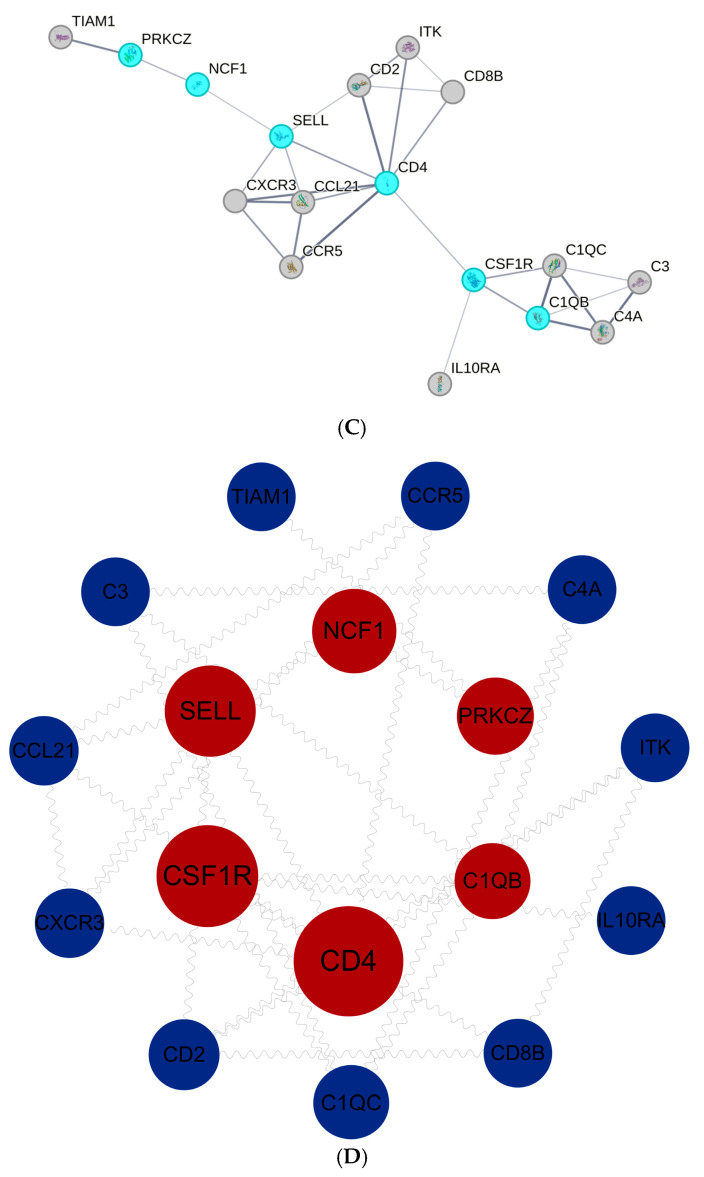
PPI network of differentially expressed genes associated with lipid metabolism and fat metabolism. Note: (**A**,**C**) Global PPI interaction networks from two comparison groups. The greater its importance in the network; the thicker the connection between nodes, the higher the confidence in the gene-gene interaction. light blue nodes highlight the regions where screened potential hub genes are located; (**B**,**D**) Corresponding core gene screening subnetworks extracted from panels (**A**) and (**C**), respectively, with red circles highlighting the screened potential key genes.

To further investigate the candidate genes regulating lipid metabolism, inflammation, and immune processes in adipose tissue, this study conducted protein–protein interaction (PPI) analysis of the DEGs listed in Table 4 and Table 5 using the STRING database. A PPI network of DEGs associated with lipid metabolism was constructed using Cytoscape software (Figure 7A), revealing interactions among 52 genes. Subsequently, the top 10 candidate genes with the highest betweenness centrality (BC) scores were identified using the cytoNCA plugin: BMP4, SOX9, WNT9A, WNT5A, PDGFRA, FGF10, PLCG2, CHRM2, PIK3CD, and RAC2 (Figure 7B). Similarly, a PPI network for DEGs related to immune processes was generated (Figure 7C), displaying interactions among 16 genes and yielding six candidate genes: CD4, CSF1R, C1QB, PRKCZ, SELL, and NCF1 (Figure 7D).

## 4. Discussion

VAT and SAT exhibit significant heterogeneity, evident not only in their anatomical locations but also in their functional characteristics and metabolic activity [12]. Previous studies have demonstrated that the risk of obesity and metabolic diseases is primarily associated with adipose tissue distribution rather than total mass [13,14,15]. This study utilized BF, GOM, and MAD samples to analyze DEGs between SAT and VAT in Sujiang pigs via transcriptome sequencing, identify key enriched pathways, and screen candidate regulatory genes involved in differential lipid metabolism. The results revealed the highest number of significantly DEGs between BF and GOM (3005 DEGs); the difference between BF and MAD was intermediate (975 DEGs); and the smallest difference occurred between GOM and MAD (892 DEGs). As a type of VAT, GOM exhibits higher metabolic activity and greater plasticity compared to BF [16,17]. In contrast, although MAD is also a form of VAT, it shares certain developmental features with subcutaneous adipose depots [18]. These findings are consistent with the observation that DEGs between BF and GOM are most numerous, whereas the number of DEGs between BF and MAD is relatively lower. Additionally, differences exist in energy metabolism and endocrine function between SAT and VAT [19]. Based on these results, it is speculated that VAT (particularly GOM) may play a more active role than SAT in regulating fat metabolism or endocrine function in Sujiang pigs.

GO enrichment analysis of DEGs indicated that genes differentially expressed between SAT and VAT were primarily enriched in biological processes such as cell surface receptor signaling pathways and the regulation of multicellular organismal processes. This finding is consistent with previous studies regarding differences in adipocyte morphology and cell size between porcine subcutaneous and visceral adipose tissues [20,21]. In contrast, DEGs among VAT samples were predominantly enriched in immune system processes, immune responses, and inflammatory responses. Previous studies have confirmed that visceral adipose tissue harbors a greater abundance of inflammation- and immune-related cells and is associated with diseases including diabetes, hyperlipidemia, and metabolic syndrome [22], which aligns with the GO enrichment findings of this study. KEGG enrichment analysis revealed that DEGs between SAT and VAT were enriched in pathways such as Rap1, Hippo, cAMP, and Wnt signaling. The Rap1 [23,24] and cAMP [25,26] signaling pathways are involved in the regulation of lipid metabolism; the Hippo signaling pathway plays a key role in adipocyte differentiation [27,28,29]; and the Wnt signaling pathway regulates adipocyte differentiation [30] as well as genes related to fatty acid oxidation, thereby affecting lipid metabolism [31]. These findings indicate that the KEGG-enriched pathways distinguishing SAT and VAT are mainly related to lipid metabolism. Conversely, DEGs within VAT were mainly enriched in pathways including complement and coagulation cascades, cytokine–cytokine receptor interaction, and chemokine signaling. The complement and coagulation cascade 2d [23,32], hematopoietic cell lineage [33], and cell adhesion molecules [34,35] are closely related to immune and inflammatory processes. Furthermore, upregulation of cytokine-cytokine receptor interaction [36] and chemokine signaling [37] can exacerbate metabolic inflammation. Overall, the KEGG-enriched pathways among VAT are mainly related to immune and inflammatory responses. In conclusion, the results of the KEGG and GO enrichment analyses are consistent. GO analysis of DEGs between SAT and VAT indicated enrichment in biological processes such as cell surface receptor signaling and the regulation of multicellular organismal processes, while KEGG analysis revealed the specific signaling pathways involved in these processes (such as Rap1, Hippo, cAMP, and Wnt) [38,39,40,41,42,43]. Similarly, GO analysis of DEGs in VAT showed enrichment in immune system and inflammatory response processes, while KEGG analysis identified enrichment in signaling pathways related to immune and inflammatory responses, such as complement and coagulation cascades, cytokine-cytokine receptor interaction, and chemokines [44,45,46]. The GO and KEGG enrichment analyses complement each other, providing a basis for the functional annotation of DEGs and the screening of candidate genes. Differences between SAT and VAT were enriched in metabolism- and development-related pathways, suggesting functional differentiation: SAT tends to be involved in lipid synthesis and storage, whereas VAT exhibits higher metabolic activity [6,12]. Additionally, the significant enrichment of immune- and inflammation-related pathways in VAT indicates that VAT may be more active than SAT in immune regulation and inflammatory responses. Previous studies have pointed out that fat distribution, rather than total mass, is a key determinant of metabolic health [13,14,15]. Based on this, it is speculated that excessive accumulation of visceral fat may be related to metabolic inflammation in pigs, thereby affecting their growth performance and health status. Therefore, further studies are needed to verify the specific roles of the aforementioned candidate pathways in depot-specific accumulation, fat immunity, and inflammation-related traits in pig adipose tissue.

Based on KEGG enrichment analysis results, this study screened significantly differentially expressed genes in pathways related to lipid metabolism and immune-inflammatory responses and conducted PPI network analysis. Ten potential candidate genes regulating lipid metabolism were identified: BMP4, SOX9, WNT9A, WNT5A, PDGFRA, FGF10, PLCG2, CHRM2, PIK3CD, and RAC2; six potential candidate genes involved in immune and inflammatory responses were also identified: CD4, CSF1R, C1QB, PRKCZ, SELL, and NCF1. Genes in the WNT signaling pathway significantly influence lipid metabolism. WNT9A has been linked to adipocyte size, abundance, and metabolic status [47]. During early obesity, WNT5A expression tends to decline with cell passaging, and WNT5A may suppress mid- and late-stage adipocyte differentiation during adipogenesis [48]. PDGFRA has been reported to be associated with the metabolic balance of peripheral adipose tissue and adipocyte function [49]. Previous studies have shown that overexpression of PDGFRA in fibro-adipogenic precursors promotes adipocyte differentiation through a Wnt-dependent mechanism [50]. WNT9A and WNT5A are enriched in the Hippo and Wnt signaling pathways, whereas PDGFRA is enriched in the Rap1 and calcium signaling pathways. The expression levels of WNT9A, WNT5A, and PDGFRA show significant differences among the BF, GOM, and MAD groups, with consistent trends: the GOM group has the highest expression, followed by the MAD group, and the BF group has the lowest. CSF1R plays a recognized role in core immune and inflammatory metabolic pathways; aberrant activation of this gene has been implicated in chronic inflammatory conditions [51]. The C1QB gene has been shown to play a significant role in antiviral immune responses [52]. CD4, a T cell surface marker, plays a central role in immune regulation [53]; notably, three SNPs in the porcine CD4 gene have been identified as potential molecular markers for disease-resistant breeding [54]. CSF1R is significantly enriched in multiple pathways, including the cytokine-cytokine receptor interaction pathway and the hematopoietic cell lineage pathway, while C1QB is enriched in the complement and coagulation cascade pathway, and CD4 is enriched in the cell adhesion molecules pathway. The expression levels of CSF1R, C1QB, and CD4 show a gradual decline from the GOM group to the MAD group and then to the BF group, with statistically significant differences between the groups. Based on the above transcriptomic analyses, these candidate genes (WNT9A, WNT5A, PDGFRA, CSF1R, C1QB, and CD4) are potentially associated with the regulation of lipid metabolism and immune-inflammatory responses in porcine adipose tissue. The limited sample size may affect the generalizability of the findings. Future studies with larger sample sizes and functional assays (e.g., cell-based experiments) are needed to validate the proposed roles of these candidate genes.

## 5. Conclusions

This study performed transcriptome sequencing on BF, GOM, and MAD of Sujiang pigs to identify differentially expressed genes and signaling pathways between visceral and subcutaneous fat depots. GO, KEGG, and PPI analyses indicate that WNT9A, WNT5A, and PDGFRA are candidate genes potentially involved in lipid metabolism, whereas CSF1R, C1QB, and CD4 may mediate immune and inflammatory responses within adipose tissue. These findings provide transcriptomic insights into the molecular regulation of adipose tissue metabolism and may inform future studies on fat deposition traits in pigs.

## Figures and Tables

**Table 1:** Primers information for RT-qPCR.

Target-Gene	Primer-Sequencing (5′–3′)	Product-Length/bp
GAPDH	F: 5′CCTGGAGAAACCTGCAAAATA3′	100
	R: 5′AACCTGGTCCTCAGTGTAGCC3′	
C1QB	F: 5′CAACGCCAATGAGAACTACGA3′	189
	R: 5′CCCGTGGTGACCTGGAAG3′	
CD163	F: 5′CTTCACTTTTGCTGTAGTCGCT3′	101
	R: 5′ACTTGTTTTCACCACCCGTTA3′	
C1QC	F: 5′TGTCTAATAGCAAGCAGGTCAGC3′	81
	R: 5′CGTTGACTCCCAGCCACAC3′	
TIMP1	F: 5′AGCCTGTACCTGCGTCCC3′	103
	R: 5′GGCGGTCTGGTTGAACTCT3′	
TFPI2	F: 5′CCGATTAGAAGTCAGTAAGAGGC3′	225
	R: 5′TAGCGAGTCACATTAGCAGAACA3′	

**Table 2:** Sequencing quality data statistics.

Sample	Raw-Reads	Clean-Reads	Error-Rate (%)	Q20 (%)	Q30 (%)	GCcont (%)
1-SJ-BF	45,328,520	43,207,246	0.00%	98.23%	93.91%	49.94%
2-SJ-BF	59,511,254	57,187,482	0.00%	98.47%	94.56%	50.27%
3-SJ-BF	48,852,256	47,061,860	0.00%	98.48%	94.61%	51.83%
1-SJ-GOM	51,741,320	49,583,022	0.00%	98.38%	94.35%	52.76%
2-SJ-GOM	67,300,442	64,788,442	0.00%	98.43%	94.48%	52.40%
3-SJ-GOM	68,261,812	65,624,900	0.00%	98.39%	94.38%	53.35%
1-SJ-MAD	54,058,838	51,653,302	0.00%	98.33%	94.18%	51.19%
2-SJ-MAD	49,516,472	47,745,568	0.00%	98.47%	94.60%	52.38%
3-SJ-MAD	57,952,476	55,544,084	0.00%	98.33%	94.22%	51.42%

**Table 3:** Sequencing data comparison results.

Sample	Total-Reads	Total-Mapped	Multiple-Mapped	Unique-Mapped
1-SJ-BF	42,072,664	40,959,127 (97.35%)	1,078,738 (2.56%)	39,880,389 (94.79%)
2-SJ-BF	56,848,248	55,523,621 (97.67%)	1,601,092 (2.82%)	53,922,529 (94.85%)
3-SJ-BF	46,518,194	45,425,522 (97.65%)	1,222,766 (2.63%)	44,202,756 (95.02%)
1-SJ-GOM	48,481,628	47,383,941 (97.74%)	1,277,086 (2.63%)	46,106,855 (95.10%)
2-SJ-GOM	63,920,116	62,283,614 (97.44%)	1,870,446 (2.93%)	60,413,168 (94.51%)
3-SJ-GOM	65,018,808	63,450,338 (97.59%)	1,864,607 (2.87%)	61,585,731 (94.72%)
1-SJ-MAD	51,354,606	50,018,678 (97.40%)	1,230,682 (2.40%)	48,787,996 (95.00%)
2-SJ-MAD	47,247,004	46,121,231 (97.62%)	1,339,398 (2.83%)	44,781,833 (94.78%)
3-SJ-MAD	55,358,130	54,168,571 (97.85%)	1,361,902 (2.46%)	52,806,669 (95.39%)

**Table 4:** Differentially expressed genes in KEGG-enriched lipid metabolism-related pathways.

Pathway Name	Gene Name	Number of Genes
Rap1 signaling pathway	CALML4, CSF1R, EGF, FGF1, FGF10, FLT4, FYB1, MET, NGFR, PDGFRA, PIK3CD, PRKCB, PRKCZ, RAC2, RASSF5, SIPA1L1, TIAM1	17
Hippo signaling pathway	WNT2B, FGF1, WNT16, PRKCZ, CCND2, WNT9A, WNT5B, BMP7, TCF7, BMP5, LEF1, AXIN2, BMP4, GDF6, WNT10A, WNT5A, FZD7	17
Calcium signaling pathway	CALML4, PTAFR, PLCG2, CACNA1D, GNA15, PTK2B, PRKCB, P2RX6, PDGFRA, ATP2A3, CHRM2	11
cAMP signaling pathway	HTR1B, CALML4, ATP1B1, TIAM1, CACNA1D, SOX9, CFTR, RAC2, GABBR2, CREB5, PIK3CD, ADCY10, CHRM2	13
Wnt signaling pathway	WNT2B, WNT16, CCND2, NKD1, WNT9A, WNT5B, TCF7, LEF1, AXIN2, RAC2, SFRP4, WNT10A, PRKCB, WNT5A, FZD7, SFRP1, GPC4, DKK1, BAMBI	19
Total (excluding duplicate genes)		54

**Table 5:** Differentially expressed genes in KEGG-enriched immune regulation pathways.

Pathway Name	Gene Name	Number of Genes
Cytokine-cytokine receptor interaction	CCL21, FLT4, BMP7, IL10RA, CSF1R, CXCR3, PDGFRA, CCR5	8
Cell adhesion molecule	NEO1, CD2, CD4, SELL, CD8B	5
Chemokine signaling pathway	CCL21, PRKCZ, TIAM1, ITK, CXCR3, NCF1, CCR5	7
Complement and coagulation cascade	C1QB, C3, C1QC, C4A, KLKB1	5
Hematopoietic cell lineage	CSF1R, CD8B	2
Total (excluding duplicate genes)		22

## Data Availability

All raw and processed datasets generated and analyzed during the current study are available from the corresponding author on reasonable request. No new public datasets were generated in this research.

## Supplementary Materials

The following supporting information can be downloaded at: https://www.mdpi.com/article/10.3390/life16061024/s1, Table S1: Top 10 differentially expressed genes between BF and GOM groups; Table S2: Top 10 differentially expressed genes between BF and MAD groups; Table S3: Top 10 differentially expressed genes between GOM vs. MAD groups.
