# Supplementary material for: Transcriptomic Profiling of Adipose Tissues in Sujiang Pigs Reveals Candidate Genes Associated with Tissue-Specific Fat Deposition
Source: Life (Basel). 2026 Jun 18;16(6):1024. doi: 10.3390/life16061024 (PMC13301325; doi:10.3390/life16061024)
Supplement: Supplementary file 1 [file life-16-01024-s001.zip › life-4326592-supplementary.pdf]

Table S1. Top 10 differentially expressed genes between BF and GOM groups

Gene id	Gene Name	log2FoldChange	*p*-Value	result
ENSSSCG00000011904	UPK1B	−10.53	9.92E-101	down
ENSSSCG00000035299	SFRP4	4.84	7.03E-91	up
ENSSSCG00000003702	GATA6	−5.72	8.62E-85	down
ENSSSCG00000033657	GREM1	−7.85	1.60E-79	down
ENSSSCG00000035859	WNT5A	−4.51	4.16E-77	down
ENSSSCG00000014117	THBS4	6.02	5.12E-76	up
ENSSSCG00000013551	C3	−6.00	7.79E-76	down
ENSSSCG00000009237	HPSE	−7.53	8.93E-76	down
ENSSSCG00000040349	HOXD8	3.08	1.52E-67	up
ENSSSCG00000038966	KRT7	-9.87	1.57E-66	down

Table S2. Top 10 differentially expressed genes between BF and MAD groups

Gene id	Gene Name	log2FoldChange	*p*-Value	result
ENSSSCG00000033657	GREM1	−8.03	9.92e-56	down
ENSSSCG00000020737	ZNRD2	−7.04	1.04e-42	down
ENSSSCG00000039161	MEIS1	−3.34	7.41e-34	down
ENSSSCG00000011717	IGSF10	−6.04	2.27e-33	down
ENSSSCG00000001570	PI16	−5.39	4.61e-32	down
ENSSSCG00000015715	EN1	4.55	3.53e-27	up
ENSSSCG00000003702	GATA6	−3.99	2.56e-26	down
ENSSSCG00000014238	PRDM6	−5.98	2.11e-25	down
ENSSSCG00000008854	CPE	−3.39	1.91e-23	down
ENSSSCG00000000917	KERA	3.69	1.10e-22	up

Table S3. Top 10 differentially expressed genes between GOM vs MAD groups

Gene id	Gene Name	log2FoldChange	*p*-Value	result
ENSSSCG00000037579	PPBP	7.33	1.02e-39	up
ENSSSCG00000007678	COL26A1	7.14	4.12e-23	up
ENSSSCG00000031694	DIRAS2	3.87	1.11e-22	up
ENSSSCG00000008141	ST6GAL2	3.86	4.53e-21	up
ENSSSCG00000030076	SLC6A13	8.39	5.08e-15	up
ENSSSCG00000017010	INSYN2B	7.56	1.42e-14	up
ENSSSCG00000029960	LRRC4B	4.23	2.6e-13	up

ENSSSCG00000029715	OLFM1	2.96	3.10e-13	up
ENSSSCG00000016174	FN1	3.06	3.32e-13	up
ENSSSCG00000030511	LGR5	5.68	5.30e-13	up
